# Retrocedent Measles

**Published:** 1889-02

**Authors:** A. M. Lawson

**Affiliations:** Georgia


### RETROCEDENT MEASLES.

By A. M. Lawson, M. D., of Georgia.

Years ago, I was called to three children in one family. They lay in a semi-comatose state, mouths open and dry, heads hot, surface elsewhere cool, pulse almost imperceptible and of countless rapidity. They with the exception of an occasional moan, lay silent, dorsal, decubitus. They had measles. The eruption had made its appearance after the usual premonitory symptoms but had receded from the surface within forty-eight hours after its first appearance; quickly followed by the above condition. With the assistance of two medical gentlemen, everything was done that is recommended in such cases; but, notwithstanding, they all died, as the other attending physicians had predicted from the outset. Which prediction I afterward found to be but the echo of the medical authorities who generally agree in stating, that such cases in young children, almost always result fatally.

The last time that measles prevailed in this place, now more than ten years ago, I was called to see I. S., eighteen months old. The patient had measles. The eruption had promptly and copiously made its appearance after the usual premonitory symytoms; but had quickly disappeared again. The patient presented the same appearance and symptoms of the cases above mentioned. I did not whip this patient with nettles, nor use the warm bath, but I tried almost everything else recommended, still my patient was not bettered, but gradually grew worse. It was on the fourth morning, I was standing by the little sufferer and saying to myself; "what shall I do next," when the following sentence recurred to my mind: "Iodide of potassium is a priceless remedy for all eucephalic diseases of childhood." I determined to test the efficacy of this statement in the case, and immediately began the use of the remedy in one third of a grain doses every two hours, directing also quite a liberal supply of water. (I forgot to mention the fact, that I had the child clothed in flannel at my first visit.) I intended seeing the child again in the after part of the day, but was prevented from doing so. On arriving at home about dusk, I found the father of the child waiting for me. "Good evening Doctor," said he, "I have come to get some more of that good physic." "The child is better then, said I. "Yes, he began to improve from the first dose." The patient continued to improve and was soon well.

The disease did not again manifest itself on the skin, but seemed to have been eliminated by the kidneys. Whether in such cases the eruption ever does, or can be restored to the surface, I am unable to say from experience, nor, have I been able to learn from others.

The use of Iodide of potassium in this connection is not mentioned in any of the authorities I have been able to consult. The fortunate and successful application of it in the above case, was the result of my seeing the above quotation mentioned in a Medical journal, some time before the case came under treatment. The article referred to was not on measles, if I am not mistaken. It was from the pen of Dr. Coldstream.

I am aware that one case does not substantiate a remedy; but from the prompt and continued beneficial effects of the remedy in this case, I feel assured it will not disappoint the practitioner in any of the complications, or sequellæ of rabeola, and I should be easily induced to try it in complicated cases of the other exanthemata, as well.

The value of a good medical Journal is incalculable. The above case is one of several fresh in my recollection in which by their aid, I have been apparently enabled to save human life. I would almost as soon attempt to practice without the materia medica, as without a good journal; for without the aid of the latter, I should often be at a loss to apply successfully the former. I have been practicing medicine thirty years, and if called upon to advise a young practitioner as to what is the chief aid to a successful practice, I should without hesitation say: Take a good medical journal. And certainly among the first duties of a practitioner is to record such facts as are calculated to benefit his professional brethren, and through them mankind at large. Otherwise while you are welcome to the recoreded xperience of others, you are not entitled thereto.

